# Prognostic role of Ki-67 in glioblastomas excluding contribution from non-neoplastic cells

**DOI:** 10.1038/s41598-021-95958-9

**Published:** 2021-09-09

**Authors:** Rikke H. Dahlrot, Julie A. Bangsø, Jeanette K. Petersen, Ann Mari Rosager, Mia D. Sørensen, Guido Reifenberger, Steinbjørn Hansen, Bjarne W. Kristensen

**Affiliations:** 1 Department of Pathology, Odense University Hospital, Odense, Denmark; 2 Department of Oncology, Odense University Hospital, Odense, Denmark; 3 Department of Clinical Research, University of Southern Denmark, Odense, Denmark; 4 Institute of Neuropathology, Heinrich Heine University, Düsseldorf, Germany; 5 Department of Pathology, Rigshospitalet, Copenhagen University Hospital, Copenhagen, Denmark; 6 Department of Clinical Medicine and Biotech Research and Innovation Center (BRIC), University of Copenhagen, Copenhagen, Denmark

**Keywords:** CNS cancer, CNS cancer, Tumour biomarkers

## Abstract

Survival of glioblastoma patients varies and prognostic markers are important in the clinical setting. With digital pathology and improved immunohistochemical multiplexing becoming a part of daily diagnostics, we investigated the prognostic value of the Ki-67 labelling index (LI) in glioblastomas more precisely than previously by excluding proliferation in non-tumor cells from the analysis. We investigated the Ki-67 LI in a well-annotated population-based glioblastoma patient cohort (178 IDH-wildtype, 3 IDH-mutated). Ki-67 was identified in full tumor sections with automated digital image analysis and the contribution from non-tumor cells was excluded using quantitative double-immunohistochemistry. For comparison of the Ki-67 LI between WHO grades (II-IV), 9 IDH-mutated diffuse astrocytomas and 9 IDH-mutated anaplastic astrocytomas were stained. Median Ki-67 LI increased with increasing WHO grade (median 2.7%, 6.4% and 27.5%). There was no difference in median Ki-67 LI between IDH-mutated and IDH-wildtype glioblastomas (*p* = 0.9) and Ki-67 LI was not associated with survival in glioblastomas in neither univariate (*p* = 0.9) nor multivariate analysis including MGMT promoter methylation status and excluding IDH-mutated glioblastomas (*p* = 0.2). Ki-67 may be of value in the differential diagnostic setting, but it must not be over-interpreted in the clinico-pathological context.

## Introduction

Patients with gliomas have different survival outcome and efforts are made to distinguish between patients with more favorable versus poor prognosis^1–4^. Methylation of the O6-methylguanine-DNA-methyltransferase (MGMT) promoter may be used to decide whether elderly glioblastoma patients will benefit from treatment with Temozolomide alone instead of radiotherapy^5–7^. Other important prognostic biomarkers for patients with diffuse astrocytic and oligodendroglial tumors include isocitrate dehydrogenase (IDH) mutation, 1p/19q codeletion and histone 3 K27M mutation^8^. These three molecular alterations have since 2016 been used as strong defining diagnostic markers for the distinction of biologically and clinically distinct glioma entities in the revised World Health Organization (WHO) classification of central nervous system tumors 2016^9^. Nevertheless, additional reliable biomarkers are urgently needed for better assessment of prognosis for the individual glioma patient—including patients having the most frequent glioma—the IDH-wildtype glioblastoma.

Ki-67 is a nuclear protein that directly reflects a specific physiological proliferative state of the cell expressing this marker^10^. Association of the Ki-67 labelling index (LI) in tumour tissues with patient survival has been reported for several cancer types^11–17^. In gliomas, immunohistochemical assessment of the Ki-67 labeling index has become the most widely used method for measuring proliferation in the diagnostic setting. Different studies have investigated the prognostic potential of Ki-67 in gliomas as reviewed by Chen et al.^18^. The Ki-67 LI increases with increasing WHO grade^19–23^ and high Ki-67 LI is associated with poor overall survival (OS) in patients with lower grade gliomas or ependymomas^24–26^. In the glioblastoma patients, the prognostic role of Ki-67 LI is less clear. Some groups showed that a high Ki-67 LI was associated with improved OS^27–29^, whereas other groups reported that high levels of Ki-67 were associated with poor OS^20,21,23,30^. Yet other groups reported that Ki-67 LI was not associated with OS^3,19,31–34^. These previous studies on Ki-67 did not include MGMT promoter methylation status and post-surgical treatment in the survival analysis although these parameters have significant prognostic impact on the outcome in the glioblastoma patients.

The reported results may also be influenced by observer-based scoring, which is prone to intra- and inter-observer variation, e.g. due to different counting methods and subjective assessment of staining positivity. Vörös et al.^35^ reported that a semi-quantitative assessment of the reproducibility of the Ki-67 LI in breast cancer is an acceptable and reproducible method. Similar approaches based on automated quantification have also been used to assess Ki-67 LI in tumors in the brain; indicating that automated digital quantification may be more robust than semi-quantitative scoring performed by pathologists^36–38^.

Another aspect influencing the results in previous studies is the Ki-67-positive non-neoplastic cells within the tumor tissue. A considerable number of microglia and macrophages, with a certain proliferative potential, can be found in brain tumors, and especially high-grade gliomas are characterized by dense infiltration with activated microglial cells and macrophages, lymphocytic infiltrates, reactive astrocytic gliosis and vascular proliferation^39,40^. Thus, non-neoplastic cells and in particular activated microglia and macrophages contribute to the overall proliferative activity and Ki-67 labeling in these tumors and may influence results obtained in earlier studies^41^. We expected that we, by exclusion of the Ki-67 contribution from proliferating non-tumor cells would be able to assess the prognostic value of the true Ki-67 level in tumor cells in glioblastomas more precisely than previously. Therefore, we used a double immunohistochemical staining approach combining Ki-67 immunostaining with a cocktail of antibodies against CD45 (immune cells), CD31 (vascular structures), Iba-1 (microglia and macrophages) and smooth muscle actin (ASMA) (vascular structures), which made it possible to identify non-neoplastic Ki-67 positive cells in the investigated glioma tissues. This allowed us to obtain a more precise evaluation of the Ki-67 LI in the actual tumor cell fraction of the tumor tissue. Because we investigated central tumor tissue only, it was decided not to stain for glial cells and neurons. To obtain a representative Ki-67 LI for each tumor we used full sections—like in daily diagnostics—instead of tissue microarrays consisting of tissue cores being only 1–3 mm in diameter.

The aim of this study was to investigate the prognostic value of Ki-67 LI restricted to glial tumor cells using a digital computer-based quantification method and a double immunohistochemical staining approach for exclusion of non-neoplastic cells. This approach was used on a well-annotated population-based cohort of astrocytic tumors. Importantly, critical prognostic parameters such as IDH mutation, *MGMT* promoter methylation as well as post-surgical therapy were taken into account.

## Materials and methods

### Patients

From a population-based cohort of 433 patients in the Region of Southern Denmark we identified 181 glioblastoma patients (178 IDH-wildtype, 3 IDH-mutated) with a sufficient amount of viable tumor tissue for immunohistochemical analyses. For the purpose of comparison, tissue from 9 IDH-mutated diffuse astrocytomas and 9 IDH-mutated anaplastic astrocytomas were stained. Tissue were obtained from the local pathological department, where it was ruinously stored. All patients underwent initial surgery between 01.01.2005 and 31.12.2009, and no treatment was received prior to surgery. This cohort has been described thoroughly and has been used in previous biomarker studies^40,42–48^. Patient characteristics are listed in Table 1.

**Table 1:** Patient characteristics. Curative intended treatment consist of radiotherapy 59.4 Gy/33 fractions with concommitant and adjuvant Temozolomide and palliative treatment is radiotherapy 34 Gy/10 fractions or chemotherapy. ECOG = Eastern Cooperative Oncology Group.

	WHO grade IV (n = 181)	Patients receiving curative intended treatment (n = 97)
Overall survival, months		
Median (range)	9 (0.03–115)	17 (3.4–115)
Dead	179 (98%)	95 (98%)
Alive	2 (2%)	2 (2%)
Age (median, range)	65 (25–82)	62 (38–81)
Gender		
Male	105 (58%)	57 (59%)
Female	76 (42%)	40 (41%)
ECOG Performance status		
0–2	114 (63%)	80 (82%)
> 2	67 (37%)	17 (18%)
Resection		
Biopsy	16 (8%)	5 (5%)
Partiel	106 (58%)	63 (65%)
Total	59 (32%)	29 (30%)
Post-surgical treatment		
None	31 (17%)	
Palliative	52 (29%)	
Curative intend	97 (54%)	
MGMT promoter status		
Methylated	72 (40%)	35 (36%)
Un-methylated	74 (41%)	51 (53%)
Missing	35 (19%)	11 (11%)
IDH status		
Mutated	3 (1%)	2 (2%)
Wildtype	178 (99%)	95 (98%)
1p/19q co-deletion		
Yes	0 (0%)	0 (0%)
No	181 (100%)	97 (100%)
Ki-67 LI		
Median (range)	24% (0–70%)	25% (0–50%)

### Immunohistochemistry

Immunohistochemical staining was carried out using the BenchMark Ultra IHC/ISH staining system (Ventana Medical Systems, Inc, AZ, USA). The primary antibody was Ki-67/ MIB-1 (monoclonal mouse antibody, Dako, no. M7240). To detect Ki-67 labeling in non-neoplastic cells a cocktail of antibodies against CD45 (monoclonal mouse antibody, Dako, no. M0701), CD31 (monoclonal mouse antibody, Dako, no. M0823), Alpha-smooth muscle actin (ASMA) (monoclonal rabbit antibody, Spring Bioscience no. M4710) and ionized calcium-binding adaptor molecule-1 (Iba1) (polyclonal rabbit antibody, Wako) was used for double immunohistochemical staining. For Ki-67 a detection system UVDAB-HRP plus amplification followed by application of the uDAB-kit (ref. no. 760–500) was used. For the other antibodies, we used the detection system UVRed-AP followed by application of the uRed-kit (ref. no. 760–501). Nuclear counter staining was performed using Hematoxylin II (Ventana Medical Systems, Tucson, AZ) at the BenchMark Ultra instrument. Finally, slides were washed, dehydrated, and coverslipped using a Tissue-Tek Film coverslipper (Sakura, Alphen aan den Rijn, The Netherlands). The slides were scanned on the Hamamatsu whole-slide scanner (Hamamatsu, Hamamatsu City, Japan).

### MGMT assessment

The MGMT promoter status was determined using pyrosequencing (MGMT Pyro kit; Qiagen, Hilden, Germany) as described by the manufacturer. Briefly; DNA was purified from 10 lm paraffin slides using QIAamp DNA FFPE Tissue kit (Qiagen), and MGMT pyrosequencing was performed according to the kit instructions. Methylation percentages at four CpG sites were measured, if one or more sites had a methylation of 10% or higher, the tumor was considered as MGMT methylated.

### Image analysis

The digital images were analyzed using the Visiopharm software module (Visiopharm, Hørsholm, Denmark). The regions of interest were manually outlined, excluding areas containing staining-artifacts, brain parenchyma, necrosis or large vessels. Sampling was performed using systematic uniform random sampling at 20 × magnification; all images were reviewed and non-tumor areas were manually outlined. Images were analyzed using an algorithm developed in the Tissuemorph APP-Control system. The algorithm identified all nuclei expressing Ki-67 and a 2.5 μm perimeter was subsequently grown around all detected nuclei. The exclusion cocktail identified non-tumor cells by a red-labelled cytoplasm, and nuclei surrounded by red-labelled cytoplasm were subsequently excluded from further analyses. It was pre-defined that only images including more than 50% viable tumor tissue were included in further analysis and that more than 5 useable images should be obtained for each tumor. Based on a sample fraction study it was shown that sampling in 10% or more of the tissue did not provide further information than sampling in 5% of the tissue (data not shown). As sampling in 10% or more was time-consuming, sampling in 5% of the tissue was chosen. Due to small amount of tissue, 27 tumors were re-sampled at a 20% fraction.

### Statistical analyses

The non-parametric Kruskal–Wallis test was used for testing the difference in Ki-67 LI between the histological grades. Overall survival (OS) was defined as time from primary surgery until death or censoring (April 1st 2018). OS wasillustrated by Kaplan–Meier plots and differences were evaluated by log-rank tests. The median Ki-67 LI was pre-defined as the cut-off value. Due to the high number of tumors, immunostainings were performed in nine runs. Each run included a positive control for Ki-67 LI estimation. Run-to-run variations were analyzed; no significant variation was detected (*p* > 0.05).

### Ethics

The study was approved by the local Committee on Health Research Ethics in Southern Denmark and the Danish Data Protection Agency. A dispensation was given by the local Committee on Health Research Ethics in Southern Denmark allowing colletion of data without informed consent from the patients. Use of the tissue was not prohibited in any of the patients according to the Danish Tissue Application Register. All methods were carried out in accordance with relevant guidelines and regulations.

### Ethics approval

The local Committee on Health Research Ethics and the Danish Data Protection Agency approved the study.

## Results

### Staining patterns and software classifier

**Figure 1:**
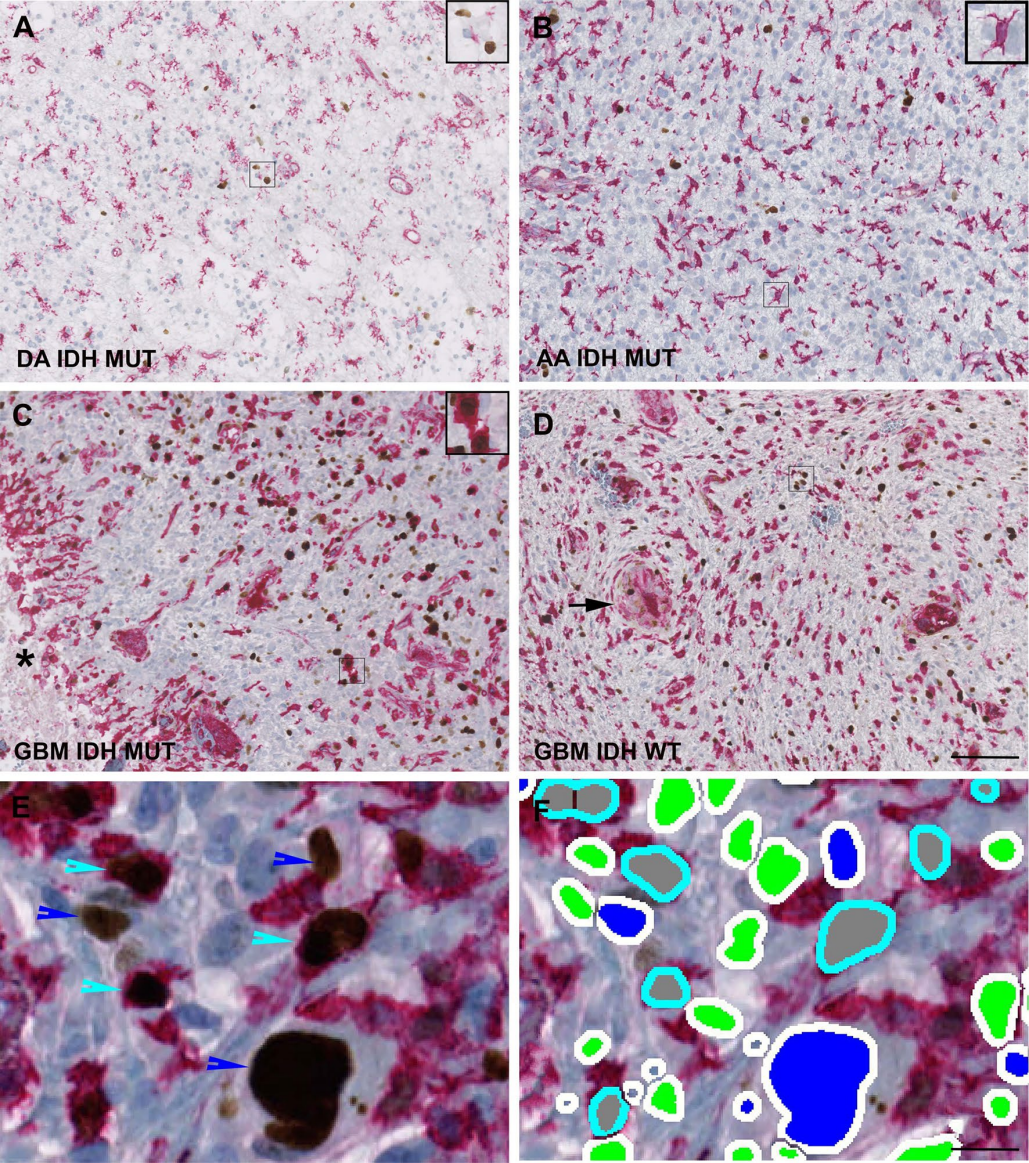
Immunohistochemical expression of Ki-67 (brown) and the exclusion-markers CD45, CD31, Iba1 and ASMA (red)—identified by a double immunohistochemical staining cocktail in astrocytic brain tumors. (**A**) IDH-mutated diffuse astrocytoma (DA) with low cellularity showing only a few scattered Ki-67 positive tumor cells not labeled by red (see insert). (**B**) IDH-mutated anaplastic astrocytoma (AA) with moderate cellularity showing higher presence of Ki-67 positive tumor cells not labeled by red. (**C**) IDH-mutated glioblastoma (GBM) and (**D**) IDH-wildtype glioblastoma with high cellularity and increased number of Ki-67 positive tumor cells not labeled by red. An increased fraction of non-tumor cells also expressed Ki-67 (see insert in C). The appearance of pseudopalisading necrosis with pink positive staining of microglia/macrophages (black asterix) and microvascular proliferations with pink positive staining of endothelial/smooth muscle cells (black arrow) are shown respectively in (**C**) and (**D**). (**E** + **F**) The staining was quantified by a software-based classifier identifying tumor cells having Ki-67 positive nuclei and not being labeled by red (blue arrowhead, blue label), non-tumor cells having Ki-67 positive nuclei and red cytoplasm (turquoise arrowhead, gray label) and supposed Ki-67 negative tumor cells not being labeled by red (green label). Magnification by × 20 (**A**-**D**) and × 80 (**E**–**F**). Scale bar indicates 100 µm (**A**-**D**), 25 µm (**E**–**F**).

The immunohistochemical double staining identified Ki-67 positive nuclei in both neoplastic and non-neoplastic cells (Fig. 1A-F). IDH-mutated diffuse astrocytomas (WHO grade II) with low cellularity had only a few scattered Ki-67 positive tumor cells and moderate expression of exclusion-marker (Fig. 1A), whereas IDH-mutated anaplastic astrocytomas (WHO grade III) with moderate cellularity showed moderate expression of the exclusion-marker (Fig. 1B). In glioblastomas (WHO grade IV) the frequency of non-neoplastic cells varied from moderate (Fig. 1C) to high (Fig. 1D).

Morphologically and immunohistochemically most non-neoplastic cells resembled activated microglia and tumor-infiltrating macrophages (Fig. 1A-D), but both small and large vessels were also identified (Fig. 1A-D). The trained software-based classifier easily detected Ki-67 positive and negative nuclei in both neoplastic and non-neoplastic cells (Fig. 1E–F).

### Ki-67 LI

**Figure 2:**
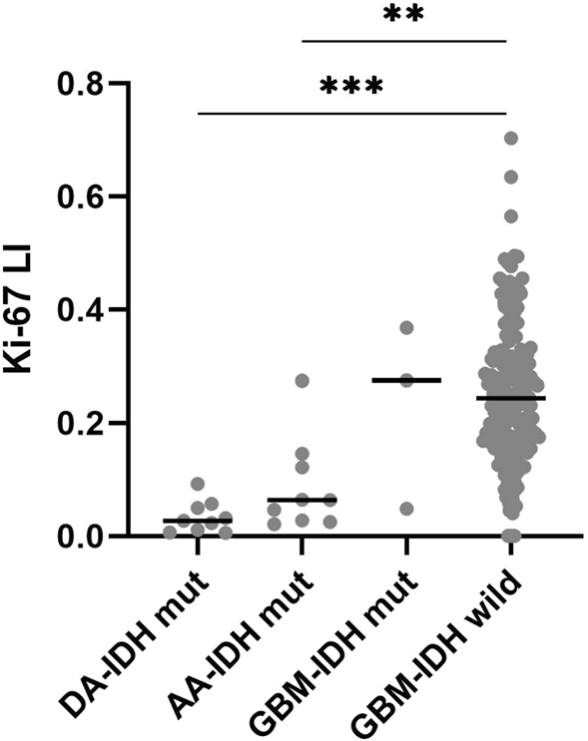
Box-plots showing the Ki-67 LI in different glioma subgroups. The horizontal line is the median.

In astrocytic IDH-mutated tumors median Ki-67 LI was 2.7%, 6.4% and 27.5% in WHO grade II, III and IV tumors. There was a significant difference between WHO grade II and IV tumors (*p* = 0.004) and between WHO grade III and IV tumors (*p* = 0.04). In IDH-wildtype glioblastomas median Ki-67 was 24.4%. This was not significantly different from IDH-mutated glioblastomas (*p* = 0.9) (Fig. 2).

**Figure 3:**
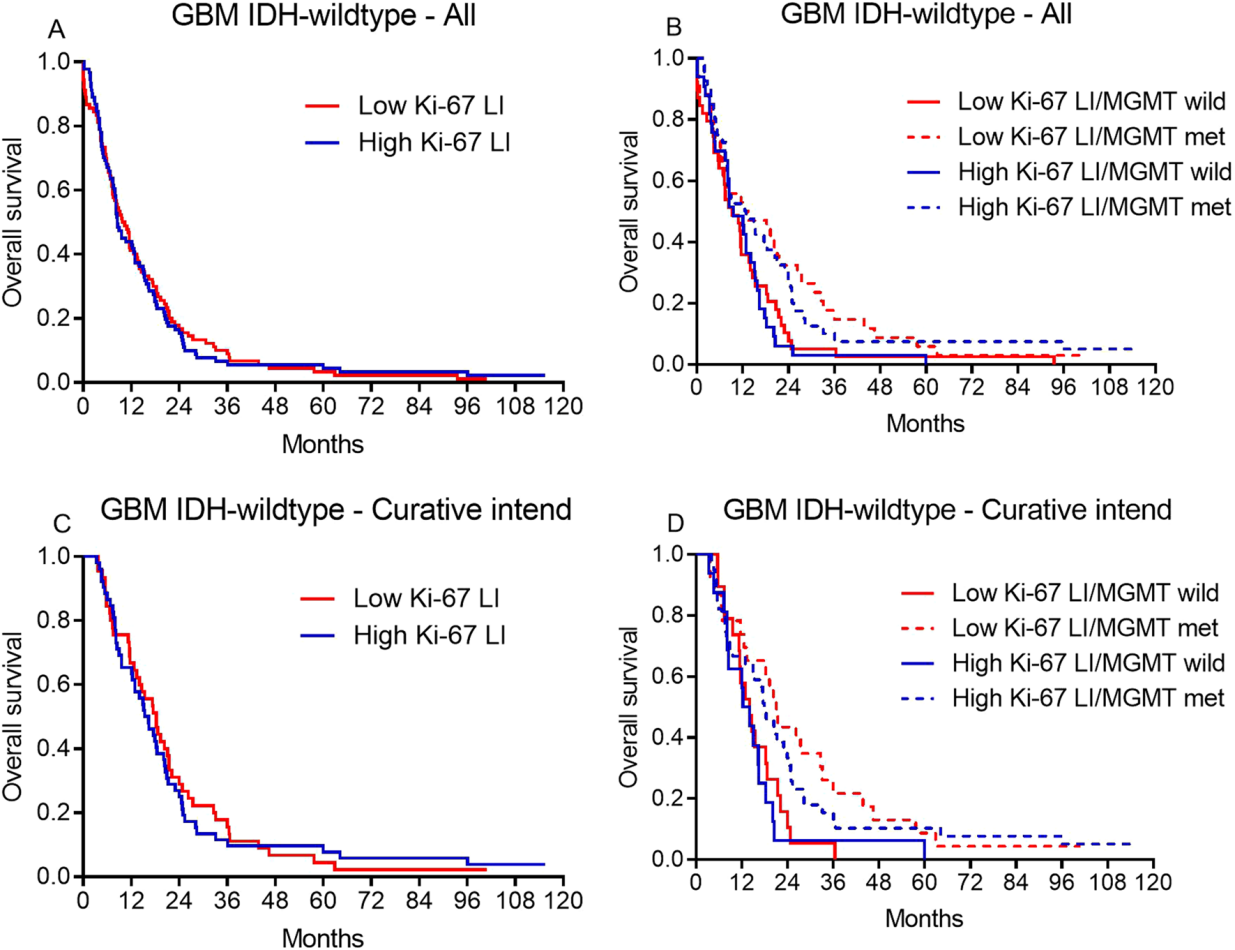
Kaplain Meier curves shown for all patients (**A**) and patients with known MGMT promoter status (**B**). Similar curves are shown for patients receiving radiotherapy 59.4 Gy on 33 fraction and concomitant and adjuvant Temozolomide in (**C**) and (**D**).

In patients with IDH-wildtype glioblastomas, median OS was 10 months in patients with low Ki-67 LI and 8 months in patients with high Ki-67 LI (*p* = 0.9). This was not significant in neither univariate (HR = 1.0, *p* = 0.9) nor multivariate analysis (HR = 1.29, *p* = 0.15) (Fig. 3A). Similar results were obtained when MGMT status was included (Fig. 3B) and when IDH-mutated glioblastomas were excluded (HR = 1.23, *p* = 0.23) (Table 2).

**Table 2:** Multivariate analysis. The first column includes all patients and second column includes all patients treated with curative intent. Curative intended treatment consist of radiotherapy 59.4 Gy/33 fraction with concommitant and adjuvant Temozolomide. Age at diagnosis is analyses as a continuous variable. HR = hazard ratio. ECOG = Eastern Cooperative Oncology Group.

Variable	All patients (n = 143)		Patients treated with curative intent (n = 95)	
	HR	*p* value	HR	*p* value
**Age at diagnosis**				
	1.02	0.005	1.02	0.04
**Gender**				
Male	1	0.09	1	
Female	0.74		0.86	0.57
**ECOG Performance status**				
0–1	1		1	
02-Apr	2.41	< 0.001	2.24	0.008
**Tumor crossing midline**				
No	1	0.002	1	
Yes	2.94		1.04	0.88
**Post-surgical treatment**				
Curative intend Palliative	1			
Palliative	1.75	0.013		
None	13.7	< 0.001		
**MGMT promoter status**				
Unmethylated	1		1	
Methylated	0.66	0.04	0.4	0.001
**Ki-67 LI**				
Low	1		1	
High	1.23	0.23	1.1	0.68

An optimal cut-point analysis was performed. It was not possible to identify a cut-point that provided more information than the median (data not shown).

In the subgroup of patients receiving treatment with curative intend (n = 97) median Ki-67 LI was 25% (0.01–50%). Ki-67 was not associated with OS in these patients in univariate (HR = 0.98, *p* = 0.9) or multivariate analyses (Table 2, Fig. 3C-D).

## Discussion

In this study we investigated the prognostic value of the Ki-67 LI in glioblastoma patients from a population-based cohort including MGMT promoter methylation and post-surgical treatment in the survival analysis. As an important methodological aspect we excluded the contribution of non-neoplastic cells to the Ki-67 LI and used digital quantification of full sections in order to minimize intra-observer bias and bias from evaluating small tumor areas, both being known pitfalls in biomarker studies^38,49,50^. Moreover, the use of full sections reflects daily diagnostics for possible glioblastomas, where most biomarkers are evaluated on full sections.

In accordance with previous results, we found that the Ki-67 LI increases with increasing WHO grade^19–23,51^. As expected, we also found that cells with microglial cell and macrophage morphology expressed Ki-67 and that the fraction of these cells increased with increasing WHO grade. This is in line with a study by Klein el al.^41^, who reported on a immunohistochemical double-labeling study with Ki-67 as proliferation-marker and Ki-M1P (CD68) as microglia marker in 40 astrocytomas WHO grade I-IV. The authors showed that proliferating microglia cells are present in all WHO grades and that the proliferative activity in microglia increased with increasing WHO grade. As the degree of microglial cell infiltration may differ between individual gliomas, we speculate that the Ki-67 contribution from microglial cells may have contributed to the divergent results reported for Ki-67 LI and outcome of glioma patients in previous studies. Therefore, we excluded the proliferative microglial cells, macrophages and other non-neoplastic cells in the tumor tissue in the present study, and specifically focused on the evaluation of proliferative tumor cells only. This is supposed to provide a more accurate estimate of the proliferative capacity of the tumor cells per se and thereby also the patient outcome.

In patients with glioblastomas, we found no correlation between Ki-67 LI and OS. This is in accordance with several other reports^3,19,31–34^, but in contrast to other studies^20,21,23,27–30^. We divided our cohort of glioblastoma patients at the median Ki-67 LI (24%), which is similar to other groups who included the contribution of Ki-67 from non-tumour cells^21,23^. Interestingly; Moskowitz et al.^31^ used the Ki-67 LI as a continuous parameter and Kuriyama et al.^34^ used quartiles, but neither of these groups identified an association between the Ki-67 LI and OS. To address a potential bias due to different cutoff values, we performed an explorative optimal cutoff analysis in our cohort; however, we were unable to identify an association between Ki-67 LI and outcome for glioblastoma patients despite the use of different cutoffs.

A strength in our study is the inclusion of MGMT promoter methylation status and information on post-surgical treatment in the multivariate analyses, as both parameters are known to influence survival. Other groups investigating the prognostic value of Ki-67 LI in glioblastomas included only MGMT status^29^, adjuvant chemotherapy^27,30,33^ or none of these important parameters in their analyses^3,20,21,23,28,31,32^. Yang et al. was the only group to^33^ include both MGMT status and post-surgical treatment in the multivariate analyses. They reported on 254 glioblastoma patients, however; no information regarding IDH-status was reported. Further; although the Ki-67 immunohistochemical stainings were performed on whole slides—scoring was performed by pathologists using a 5-point scale. Despite these methodological differences compared to our study, the authors reported that Ki-76 LI was not associated with OS, a result similar to ours. It should be noticed that all studies reporting that Ki-67 LI is associated with OS, a finding that we could not validate in our population-based patient cohort, lack information of MGMT promoter methylation status and post-surgical treatment in the multivariate analyses. Therefore, the prognostic value of Ki-67 LI in glioblastomas is most likely very limited.

Several groups, including our group^38^, have reported that the reproducibility of Ki-67 LI varies between studies. This may be due to inter- and intra-observer variability when detecting Ki-67 LI in tumors^35,50^. Polley et al. investigated intra- and inter-laboratory variability in 100 breast cancer patients between 8 different laboratories. Each laboratory scored Ki-67 as percentage of positively stained invasive tumor cells using its own method. The authors found high intra-laboratory reproducibility, whereas the inter-laboratory reproducibility was modest. In contrast to the present study, Polley et al. investigated manual scoring only. Our group has recently reported that manual scoring has limited reproducibility in glioma patients^38^, and we thus decided to use digital quantification in the present study.

## Conclusion

We found no association of Ki-67 LI with overall survival in IDH-wildtype glioblastomas, independent of the employed cutoff values and even when MGMT promoter methylation status and postsurgical treatment were included in the survival analysis. Due to the small number of IDH-mutated glioblastomas it is not possible to assess the prognostic value of Ki-67 in these patients. For the first time the prognostic value of Ki-67 was addressed using digital quantification of full sections excluding Ki-67 positive nuclei from non-neoplastic cells. Over-interpretation of the prognostic potential of Ki-67 LI in the clinico-pathological setting should therefore be avoided.

## Data Availability

Data is available from the corresponding author upon reasonable request.
